# Encoded Library Synthesis Using Chemical Ligation and the Discovery of sEH Inhibitors from a 334-Million Member Library

**DOI:** 10.1038/srep10916

**Published:** 2015-06-10

**Authors:** Alexander Litovchick, Christoph E. Dumelin, Sevan Habeshian, Diana Gikunju, Marie-Aude Guié, Paolo Centrella, Ying Zhang, Eric A. Sigel, John W. Cuozzo, Anthony D. Keefe, Matthew A. Clark

**Affiliations:** 1 X-Chem Pharmaceuticals, 100 Beaver St., Waltham, MA, USA; 2 Novartis Pharma AG, Institutes of Biomedical Research, Basel, Switzerland

## Abstract

A chemical ligation method for construction of DNA-encoded small-molecule libraries has been developed. Taking advantage of the ability of the Klenow fragment of DNA polymerase to accept templates with triazole linkages in place of phosphodiesters, we have designed a strategy for chemically ligating oligonucleotide tags using cycloaddition chemistry. We have utilized this strategy in the construction and selection of a small molecule library, and successfully identified inhibitors of the enzyme soluble epoxide hydrolase.

In the past decade, DNA-encoding of small molecule libraries has emerged as an attractive strategy for the discovery of novel ligands to biological targets^1^,^2^,^3^,^4^. Due to advances in the throughput of DNA-sequencing, DNA-encoding allows the interrogation of vast numbers of compounds, exceeding by orders of magnitudes the capacity of traditional “one compound per well” screening approaches^5^,^6^,^7^,^8^,^9^,^10^. Encoding strategies can be divided into two categories: DNA-directed methods, in which synthetic chemistry is programmed by DNA complementarity; and DNA-recording methods, in which the encoding oligonucleotide is built during the library synthesis, so that the synthetic history of each molecule is recorded in its DNA strand.

DNA-recording approaches require the iterative construction of both the chemical library members and the encoding oligonucleotide. In the original reports of DNA-encoding from the early 1990’s, the encoding DNA was built using solid-phase oligonucleotide synthesis with phoshoramidite building blocks^11^,^12^. In 1995, however, Kinoshita and Nishigaki introduced the concept of the enzymatic ligation of encoding oligonucleotide “tags”^13^. They showed how iterative ligation could be employed to create an oligonucleotide that encodes successive combinatorial steps. Since that time, the various groups operating DNA-recorded technology have reported enzymatic construction of the DNA; both ligase- and polymerase-based methods have been described^14^,^15^.

Recent reports from Brown, El-Sagheer and Tavassolli have demonstrated that oligonucleotides containing a triazole linkage in place of a phosphodiester are competent substrates for PCR and thus could provide a “readable” encoding sequence^16^,^17^,^18^,^19^. Based on these results, and our prior experience with Cu-catalyzed alkyne-azide cycloaddition (CuAAC) of oligos^20^, we wondered whether a readable chemical ligation strategy might offer some advantages over the current enzymatic methods. Chemical ligation using CuAAC might offer more flexibility in terms of ligation conditions and sequence design, since CuAAC is a famously robust reaction and sticky ends would not be needed.

Previous work had qualitatively shown that triazole-containing oligonucleotides could be amplified by PCR and that a triazole-containing plasmid could be translated in living bacteria^17^,^18^ or human cells^19^; direct quantitation of read-through efficiency was not reported. For library encoding purposes, the most efficient possible read-through is required, as the sampling depth of the selection output will be directly dependent on the read-through efficiency. An inefficient read-through process would lead to over-weighting of the few “lucky” sequences that were successfully extended and/or amplified early in the PCR. We sought to quantify the read-through yield and determine the proportion of the “stalled” product, if present. Preliminary results using denaturing gel electrophoresis and a fluorescently labeled primer gave inconsistent results. We could not rule out incomplete denaturation as the source of fluorescent signal in high MW bands. Therefore, we designed the scheme shown in Fig. 1. The advantage of this approach was the removal of the triazole-linked template strand, which simplified subsequent LCMS analysis of the primer extension products.

Oligonucleotide **3** (see Table 1) was synthesized using a CuAAC ligation followed by biotin labeling of the 5′-amine linker (Fig. 1). We designed a Cy-5 labeled 17-mer primer **4** that was complementary to the 3′-terminal region of **3** and could undergo extension by a DNA polymerase. A 20 base region separated the end of the primer and the triazole linkage. If the DNA polymerization reaction stopped at the triazole, the primer extension reaction would add 20 nucleotides, whereas successful read-through would add 37 nucleotides.

With oligos **3** and **4** in hand, we set out to assess various DNA polymerases for their ability to read through the triazole linkage. The “stalled” product **5** and the full length product **6** were easily resolved by HPLC and identified on the basis of their molecular weights. Several DNA polymerases were examined and the ratio of **5** to **6** was used as an indicator of the translocation efficiency through the triazole linkage. As a positive control, we also conducted primer extension reactions using the template **7** that contained only natural phosphodiester linkages. The results are illustrated in Fig. 1 and summarized in Table 2. Of the polymerases studied, we observed that Klenow fragment was the most efficient at reading through the triazole linkage, with ca. 80% of the extended primer product being of full length. Interestingly, while the mass spectrum of **6** showed clean formation of the full-length material, the peak corresponding to **5** actually showed a mixture of species. Masses corresponding to **5** (11,880 Da), **5** – A (11,567 Da) and to a lesser extent, **5** + A (12,192 Da) were observed. While the mechanism of formation of these side products is unknown, the fact that similar products were not observed in the full-length product indicates that they are a consequence of triazole-induced stalling during the polymerization reaction. We wondered if the 20-base sequence upstream of the triazole could be reduced in length while maintaining read-through. Oligonucleotide **8**, in which the run-up length was reduced to 8 bases, was synthesized. Read-through experiments with Klenow fragment gave similar results as with 3.

The conceptual design of the chemical ligation strategy is shown in Fig. 2. Tag sequences contain both azide and alkyne functional groups to allow for iterative ligation. To minimize constant regions and thus tag length, we chose to pursue a non-templated ligation strategy. To prevent self-reaction of the tags, we turned to a chemical protection strategy for the alkyne using the alkynyl-TIPS group. Removal of the TIPS group from alkyne-containing oligonucleotides had been shown previously^21^. Since non-thermostable Klenow was the most efficient polymerase for read-through, sequencing of the library would require separate primer extension and PCR amplification steps. In our hands, library templates with 4 triazole junctions could not be amplified by PCR without first undergoing Klenow extension (See Supporting Information).

Synthesis of 3′-TIPS protected oligos required the TIPS-containing monomer **11**. This material was obtained from Prime Organics Inc. (Woburn, MA). With monomer **11** in hand, we set out to obtain a set of tag oligonucleotides for library synthesis. Tag oligonucleotides were synthesized by Biosearch Technologies (Novato, CA) and designed to conform to the following architecture:

5′-azido-TXXXXXXXXXXXXXU-3′-propargyl-TIPS

With the azido / TIPS propargyl tags in hand, we were able to construct a template containing multiple triazole junctions, similar to a theoretical library member. Oligo **9** was constructed by a repetitive sequence of CuAAC ligation and TIPS deprotection. It was used as a template for primer extension as described above using the FAM-labeled version of primer **4**. The results of the primer extension reaction are shown in Fig. 3. The major component was again the full-length product. In this case, however, the mass spectrum of the product showed a single species with a molecular weight of 21,795 Da, a loss of approximately an adenosine residue from the expected mass of 22,110 Da. Peaks corresponding to truncated products arising from termination at the first and second triazole junctions were also observed, with multiple products apparent, including adenosine deletions. The previous experiments utilizing a single triazole linkage indicated that the rG^tr^T junction was correctly transcribed by Klenow fragment. In this case there are both the rG^tr^T and(r)U^tr^T junctions in the template; we hypothesize that the latter is copied into a single A residue. Similar behavior was observed by El-Sagheer and Brown in transcribing across T^tr^T junctions, although the linkage chemistry was different than that used here^16^. In a later report using the identical linkage as the present work but with different polymerases (Go*Taq* and *Pfu*), no skipping of T residues was observed^17^.

In addition to the experiments described above, we extended the study to template **10** containing three triazoles. Primer extension of this template again gave acceptable yields (>50%) of full-length product. In this case, the mass of the full length product corresponded to loss of two A residues, consistent with the behavior observed with template **9**. These data gave us confidence that DNA encoding regions containing multiple triazole linkages would be readable by primer extension and sequencing. While the possibility of skipped T’s at the junctions might complicate the sequence analysis, we reasoned that as long as these nucleotides were not used to encode information, losing an A residue from the junctions was tolerable. We chose to embark on synthesis of a chemically DNA-encoded small molecule library.

For library synthesis, we chose a strategy based on a set of bromoarylcarboxylic acids. The synthetic route is shown in Fig. 4. In the first cycle of library synthesis, the starting material was split into 2,259 wells for tagging by CuAAC. After chemical ligation, 2,259 primary amines were installed onto the aldehyde by reductive amination. After pooling the wells, the TIPS group was removed by treatment with TBAF. In the next diversity-generating step, the material was split into 666 wells, tagged by CuAAC, and then acylated with 3 × 222 bromoarylcarboxylates. After pooling and deprotection, a final diversification step was conducted with 669 tags and 667 boronic acid / boronate ester building blocks, which were installed by Suzuki cross-coupling (the extra tags were used to encode the products of reaction failure). Installation of boronates onto oligonucleotides by Suzuki coupling has been shown previously^22^,^23^, including in the context of encoded libraries^24^. We adapted procedures commonly used for Suzuki coupling under aqueous conditions^25^,^26^,^27^. After completion of the final step, the wells were pooled and the library purified by reverse-phase HPLC. The recovered yield of library after HPLC purification following the Suzuki chemistry was 3-fold lower than that in the previous two steps (17%, compared to 53% and 61% respectively). We believe the loss is related to the lower yield of the Suzuki reaction and the generally lower recovery of library DNA we’ve observed after transition-metal mediated chemistries. Whatever the source of the losses, they do not appear to arise from the chemical ligation protocol, as they were not observed in the first two synthesis cycles.

Prior to selection, the 334 million compound library was subjected to TIPS deprotection and ligated by CuAAC with a terminal self-priming hairpin oligonucleotide. Primer extension with Klenow yielded library with a double-stranded encoding oligonucleotide, suitable for affinity selection. We subjected the library to affinity-mediated selection against the target soluble epoxide hydrolase (sEH). This enzyme is involved in the conversion of epoxyeicosatrienoic acids (EETs) to dihydroxyeicosatrienoic acids (DHETs). Various studies have shown that EETs have anti-inflammatory properties, and so it has been suggested that inhibitors of sEH could have beneficial effects in COPD, cardiovascular disease, and even diabetes. Researchers at GSK have reported on sEH inhibitors that show beneficial effects in a cigarette smoke exposure mouse model^28^,^29^. These compounds were derived from leads originally discovered using a DNA-encoded library platform.

Selections were performed by conventional methods as described elsewhere. Library was incubated with His-tagged sEH protein in solution, followed by protein capture using an immobilized-metal affinity chromatography matrix (IMAC) resin. After washing, bound library members were eluted by heat denaturation. The resulting population of enriched library members was subjected to a second round of selection. The selection output was amplified by PCR and sequenced using an Illumina HiSeq 2500 instrument; the sequencing output was translated into chemical identifiers.

The output of the sEH selection was visualized using a cubic plot. Several families of compounds were enriched, with the most abundant being the one illustrated in Fig. 5. The family clustered into two well-defined planes, each one corresponding to a particular boronate building block. The two building blocks were obviously related by virtue of their *m*-boronyl benzamide substructure. Interestingly, the N-benzyl carboxamide moiety had been previously shown to be a key component of sEH inhibitors discovered through an encoded library appoach. Sequencing of naïve library and library selected without sEH did not show these features, giving us confidence that these compounds were specifically binding to sEH protein.

The prevalence of the individual building blocks in the family is shown in Table 3. Cycle A amines showed little structural specificity. No single amine was present in more than 0.7% of the family members. The cycle B bromoarylcarboxylates also showed variation, but with obvious structural trends. The three most abundant Cycle B building blocks in the family were *p*-brominated nicotinates or picolinates. The most abundant trisynthon in the family was the combination of N-aminoethyl pyrazole at Cycle A, 5-bromonicotinic acid at Cycle B, and N-cyclohexyl-3-boronylbenzamide at Cycle C. The corresponding compound **12** (with a methyl group in place of the DNA linker) was synthesized and assayed for sEH inhibition, and found to have an IC_50_ value of 2 nM. The building block combination that comprises compound **12** was observed thirteen times among the 147,885 sequence reads generated for the selected library, and not at all in the 2,037,412 sequence reads generated for the naïve library. This represents an approximately 30,000-fold enrichment over two affinity-mediated selection steps when compared to the average within the naïve library.

In summary, we have demonstrated that chemical ligation is a feasible approach to the catenation of encoding oligonucleotides in DNA-encoded chemistry. The method relies on the ability of Klenow fragment of DNA Polymerase I to translocate through triazole linkages in a DNA backbone. We have developed a protecting group strategy based on the alkynyl TIPS group that allows for repetitive and specific installation of multiple oligonucleotide tags. The chemical ligation strategy has been validated by the construction of a 334 million-member library of biaryls, and its successful interrogation for inhibitors of the enzyme soluble epoxide hydrolase. We believe this chemical ligation methodology represents an advance over previous methods^30^ and will expand the scope and diversity of chemistry addressable using DNA-encoded library synthesis.

## Additional Information

**How to cite this article**: Litovchick, A. *et al.* Encoded Library Synthesis Using Chemical Ligation and the Discovery of sEH Inhibitors from a 334-Million Member Library. *Sci. Rep.* **5**, 10916; doi: 10.1038/srep10916 (2015).

## Supplementary Material

Supplementary Information

## Figures and Tables

**Figure 1:**
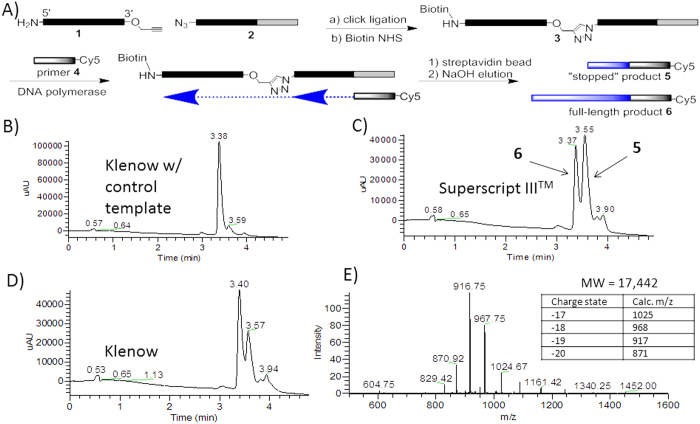
Study of polymerase read-through of single triazole junction. **A**) Scheme for substrate synthesis and read-through study. **B**) LC trace of control read-through experiment using Klenow with wild-type template. UV detection at 495 nm. **C**) LC trace of read-through products using Superscript III^TM^. **D**) LC trace of read-through using Klenow fragment. **E**) Mass spectrum of major peak in trace **D**).

**Figure 2:**
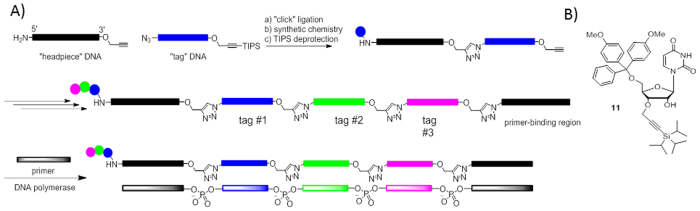
**A**) Scheme of a chemical ligation–based library synthesis strategy; **B**) Structure of compound **11**.

**Figure 3:**
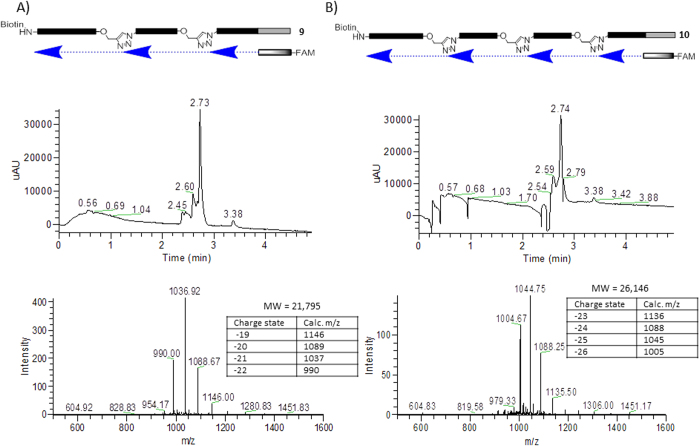
Study of Klenow read-through using FAM-labeled primer. **A**) Scheme and LCMS data for read-through of double triazole template **9**. UV detection at 495 nm. **B**) Scheme and LCMS data for read-through of triple triazole template **10**.

**Figure 4:**
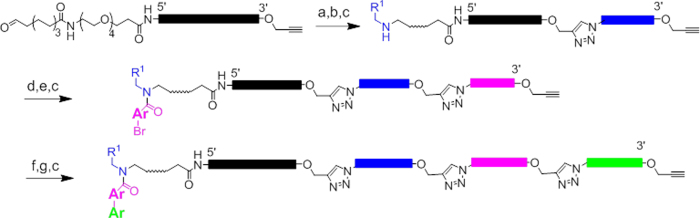
Scheme for encoded library synthesis. **a**) 2,259 5′-azido-3′-TIPS propargyl tags, Cu(OAc)_2_, sodium ascorbate, TBTA; **b**) 2,259 primary amines, NaBH_3_CN, 60 deg. C; **c**) TBAF; d) 666 5′-azido-3′-TIPS propargyl tags, Cu(OAc)_2_, sodium ascorbate, TBTA; e) 3 × 222 bromoaryl acids, DMT-MM f) 669 5′-azido-3′-TIPS propargyl tags, Cu(OAc)_2_, sodium ascorbate, TBTA; g) 667 boronic acids and esters, Pd(PPh_3_)_4_, CsCO_3._ Remaining 2 wells were encoded nulls, and received no reagent or palladium catalyst and no boronate, respectively.

**Figure 5:**
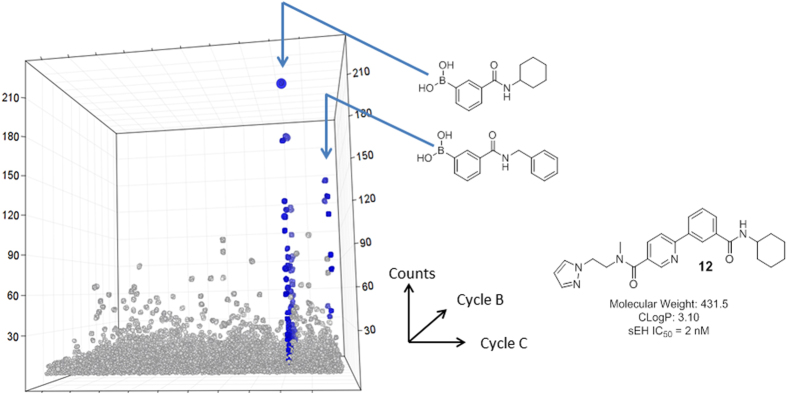
Visualization of library population after selection against sEH. The family indicated in blue was defined by two structurally related boronates. Compound **12** was designed as an exemplar of the indicated family.

**Table 1:** Oligonucleotides used in this study.

**Oligo**	**Sequence**	**Mol. Wt.**
**1**	5′-(NH2-C_6_)-TCG AAT GAC TCC GTA TrG-3′-Propargyl	5,732
**2**	5′-azido-TAT AGC GCG ATA TAC ACA CTG GCG AGC TTG CGT ACT G	11,414
**3**	5′-Biotin-PEG4 -CONH-C_6_-TCG AAT GAC TCC GAT ATrG^tr^ TAT AGC GCG ATA TAC ACA CTG GCG AGC TTG CGT ACT G	17,619
**4**	5′-Cy5-CAG TAC GCA AGC TCG	5,095
**5**	5′-Cy5-CAG TAC GCA AGC TCG CCA GTG TGT ATA TCG CGC TAT A	11,882
**6**	5′-Cy5-CAG TAC GCA AGC TCG CCA GTG TGT ATA TCG CGC TAT ACA TAT CGG AGT CAT TCG A	17,443
**7**	5′-Biotin-PEG4 -CONH-C_6_-TCG AAT GAC TCC GAT ATrG TAT AGC GCG ATA TAC ACA CTG GCG AGC TTG CGT ACT G	17,811
**8**	5′-Biotin- PEG4 -CONH-C_6_-TCG AAT GAC TCC GAT AT rG^tr^TAC ACA CTG GCG AGC TTG CGT ACT G	13,463
**9**	5′-Biotin-TEG-spacerC_3_-spacerC_3_-TCG AAT GAC TCC GAT AT rG^tr^T ATG CGT ACA GTC C U^tr^T ATA GCG CGA TAT ACA CAC TGG CGA GCT TGC GTA CTG	22,235
**10**	5′-Biotin-TEG-spacerC_3_-spacerC_3_- TCG AAT GAC TCC GAT AT rG^tr^T ATG CGT ACA GTC C U^tr^T ATG CGT ACA GTC C U^tr^T ATA GCG CGA TAT ACA CAC TGG CGA GCT TGC GTA CTG	26,848

Superscript “tr” denotes a triazole linkage. Ribose-containing residues are indicated with “r”.

**Table 2:** Read-through efficiencies of various DNA polymerase enzymes.

**Enzyme**	**Ratio of 5:6**
*E. coli* DNA Pol I	3:7
*E. coli* DNA Pol I Klenow fragment	2:8
Therminator™	4:6
Superscript III™	6:4
9°N™	6:4

**Table 3:**
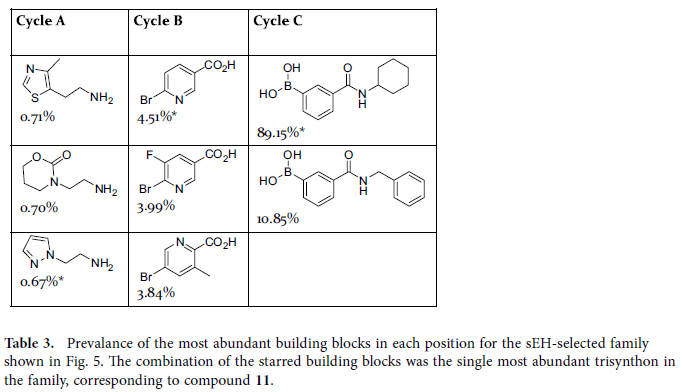
Prevalance of the most abundant building blocks in each position for the sEH-selected family shown in Fig. 5.
