# Supplementary material for: Encoded Library Synthesis Using Chemical Ligation and the Discovery of sEH Inhibitors from a 334-Million Member Library
Source: Sci Rep. 2015 Jun 10;5:10916. doi: 10.1038/srep10916 (PMC4603778; doi:10.1038/srep10916)
Supplement: Supplementary Information [file srep10916-s1.doc]

Encoded Library Synthesis Using Chemical Ligation and the Discovery of sEH Inhibitors from a 334-Million Member Library

Alexander Litovchick, Christoph E. Dumelin, Sevan Habeshian, Diana Gikunju, Marie-Aude Guié, Paolo Centrella, Ying Zhang, Eric A. Sigel, John W. Cuozzo, Anthony D. Keefe and Matthew A. Clark

Supporting Information

**Preparation of triazole-linked oligonucleotides for DNA polymerase read-through studies**

**Oligo 1**

**Oligo 2**

**Tag S4**

General protocol for chemical ligation *via* Click reaction:

The Cu (I) catalyst was prepared by mixing Cu(OAc)_2_ (10 mM in DMF), sodium ascorbate (20 mM in H_2_O) and TBTA (10 mM in DMF) in 2:2:1 ratio by volume. To a PCR tube was added 13 μL pH 7.0 phosphate buffer (500 mM in H_2_O), 1 μL of ssDNA-alkyne (10 mM in H_2_O, 10 nmol, 1 eq) and 1 μL Tag-N_3_ (10 mM in H_2_O, 10 nmol, 1 eq). To this solution was added 5 μL of Cu (I) catalyst (2 eq of Cu(II), 4 eq of sodium ascorbate, 1 eq TBTA). The reaction was incubated at room temperature overnight. After analysis by LC-MS to confirm the completion of the reaction, the DNA product was precipitated using the standard ethanol precipitation protocol (10% volume of 500 mM aq. NaCl and 3 volumes of cold ethanol). The pellet was recovered and lyophilized to dryness.

General protocol for deprotection of TIPS:

To a PCR tube was added 1 μL of DNA-alkyne-TIPS (10 mM aq. solution, 10 nmol), and 20 μL of 500 mM TBAF in DMF. The reaction was heated at 60^o^C for 2 hours when the completion of the reaction was confirmed by LC-MS. The DNA product was precipitated using the standard ethanol precipitation protocol.

**Scheme 1: One Click Junction**

**S1:** To a solution of 5 μL of **1** (10 mM in H_2_O, 50 nmol) in 45 μL of pH9.4 borate buffer (250 mM in H_2_O), was added 10 μL of Fmoc-OSu (250 mM in ACN). LC-MS showed clean conversion to product (**S1**). The DNA product was precipitated with NaCl and cold ethanol. The pellet was dissolved into 10 mM aqueous solution for the next step without purification. Expected MS: 5954; MS found: 5953.

**S2:** The general protocol for Click ligation was followed. The DNA product was precipitated with NaCl and cold ethanol. The pellet was recovered, dissolved in H_2_O, and subjected to LC purification. The fractions were collected and lyophilized to dryness. Expected MS: 17368; MS found: 17367.

**S3:** To a solution of **S2** in 200 μL of H_2_O, was added 10 μL of piperidine. The reaction was allowed to proceed at room temperature until LC-MS showed the completion. The DNA product was precipitated with NaCl and cold ethanol. The reaction yield was determined to be 34% for 3 steps from **1**, using OD (optical density) on Nanodrop. Expected MS: 17146; MS found: 17145.

**2:** To a solution of **S3** in 18 μL of pH9.4 buffer, was added 2 μL of 250 mM biotin-NHS in ACN. The reaction was allowed to proceed at room temperature for 2 hours when LC-MS showed clean conversion to the desire product. Expected MS: 17619; MS found: 17621.

**Scheme 2: Two Click Junctions**

**S5:** The general protocol for Click ligation was followed using oligo **S1** and tag **S4** as starting materials. The DNA product was precipitated with NaCl and cold ethanol. The pellet was recovered, dissolved in H_2_O, and subjected to LC purification. The fractions were collected and lyophilized to dryness. Expected MS: 10719; MS found: 10719.

**S6:** Compound **S6** was prepared from **S5** following the general protocol for deprotection of TIPS. The Fmoc protecting group was also removed under the reaction conditions. The resulting DNA product **6** was precipitated with NaCl and cold ethanol, and spin filtered (Amicon Ultra, 3K) prior to being used for the next step. Expected MS: 10341; MS found: 10342.

**S7:** Compound **S7** was prepared from **S6** using the general protocol for Click ligation with oligo **2** as ligation partner. The resulting DNA product was precipitated with NaCl and cold ethanol. The pellet was recovered, dissolved in H_2_O, and used for the next step without further purification. Expected MS: 21755; MS found: 21752.

**9:** To a solution of **S7** in 18 μL of pH9.4 buffer, was added 2 μL of 250 mM biotin-NHS in ACN. The reaction was allowed to proceed at room temperature for 2 hours when LC-MS showed complete conversion to the desire product. Expected MS: 22228; MS found: 22227.

**Scheme 3: Three Click Junctions**

The three-click oligo **S8** was synthesized in a manner identical to that of the two-click oligo **9** above. Two successive click ligations of oligo **S4** were performed prior to ligation with the terminal oligo **2.**

**Library Synthesis**

Materials

DNA headpiece and DNA tags were acquired from Biosearch Technologies, Novato, CA. Chemical building blocks (primary amines; aryl amines; bromoaryl carboxylic acids; boronic acids and esters) were all obtained from commercial sources. Primary and aryl amines were dissolved in 1:1 water/acetonitrile. Bromoaryl carboxylic acids and boronic acids/esters were dissolved in dimethylacetamide (DMA). All other reagents were purchased from Sigma-Aldrich or Acros Organics and used without any further purification.

Installation of chemical spacer

To a solution of 3’-TIPS-propargyl DNA headpiece (100 µmol, in 100 mL of 250 mM pH 9.5 borate buffer) was added 50 equivalents of Fmoc-15-amino-4,7,10,13-tetraoxapentadecacanoic acid (25 mL, 200 mM solution in DMA). To this mixture was added 40 equivalents of 4-(4,6-dimethoxy-1,3,5-triazin-2-yl)-4-methylmorpholinium chloride (DMT-MM) (10 mL of a 400 mM stock in H_2_O). The reaction was allowed to proceed for 30 minutes, then was precipitated by addition of 5 M NaCl (10% total reaction volume) and 3 volumes of cold ethanol, followed by centrifugation and lyophilization. The dry pellet was Fmoc deprotected in 100 mL 10% v/v aqueous solution of piperidine for 30 minutes, then precipitated using the same procedure above.

TIPS Alkyne deprotection

Fmoc deprotected HP-spacer (100 µmol) was dissolved in 10 mL water, and 200 mL of a tetrabutylammonium fluoride solution (500 mM in DMF) was added. This was heated at 60° C for 1 hour, then diluted with 200 mL of 2.5 M NaCl solution. Precipitation and centrifugation afforded the unprotected alkyne-containing headpiece, which was then desalted using tangential flow filtration (TFF, first washed with 200 mL of 2.5 M NaCl, then 500mL H_2_O).

Installation of dimethylacetal functionality

A 200 mM solution of sodium-9,9-dimethoxynonanoate (1.2 g, 50 eq) was made in 25 mL 1:1 DMA/H_2_O. This was then added to the TIPS and Fmoc deprotected headpiece material (100 µmol) in 100 mL of 250 mM pH 9.5 borate buffer, followed by 50 equiv, of DMT-MM as a 200 µM aqueous stock and allowed to react overnight. The completed reaction was precipitated by the procedure described above, then centrifuged and lyophilized. The material was then desalted by centrifugal filtration.

Cycle A

A 1 mM solution of dimethyl acetal functionalized headpiece (98 µmoles, 98 mL) in 200 mM pH 7.0 phosphate buffer was split into 2,259 wells (43 nmoles/well). To each well was added 1.2 equivalents of a tag (1 mM in H_2_O, 51.6 µL). Stocks of Cu(OAc)_2_ (10mM DMF, 20 mL), sodium ascorbate (20 mM water, 20 mL), and Tris[(1-benzyl-1*H*-1,2,3-triazol-4-yl)methyl]amine (TBTA, 10 mM DMF, 10 mL) were then prepared. These stocks were combined and 21.5 µL of the mixture was added to each well (2 eq Cu(OAc)_2_/well, 4 eq ascorbate/well, 1 eq TBTA/well) and the reactions were allowed to proceed overnight. Reaction progress was monitored by LCMS. Wells were then precipitated using the standard procedure, centrifuged, then allowed to air dry. The pellets were dissolved in 2% v/v acetic acid in water and heated at 50° C for 45 minutes to afford the deprotected aldehyde. The wells were then precipitated, and the resulting pellets were dissolved in 43 µL of pH 5.5 phosphate buffer (500 mM), and 80 equivalents of 1 of 2,259 primary and aryl amines (200 mM 1:1 MeCN/H_2_O, 17.2 µL/well) were added. This was followed by addition of 80 equivalents of sodium cyanoborohydride (400 mM DMF, 8.6 µL/well), then heating at 60° C for 18 hours.

Excess headpiece-alkyne was capped using 20 equivalents of benzyl azide (200 mM DMF, 4 mL) and 20 mL of the copper premix solution used in ligation. Unreacted aldehyde (library 1 mM in pH 5.5 phosphate buffer) was capped using 20 equivalents of piperidine (200 mM in 1:1 MeCN/H_2_O, 10 mL) and 20 equivalents of NaCNBH_3_ (400 mM DMF, 5 mL) at 60° C overnight. The library was then purified on reverse phase HPLC to yield 52 µmoles of material. The purified material was dissolved in 5.2 mL water, and 104 mL of TBAF solution (500 mM DMF) was added. After heating at 60° C for 1.25 hours, the DNA was precipitated, then desalted using centrifugal filtration.

# Cycle B

A 1 mM solution of Cycle A material (46.52 µmoles, 46.52 mL) in 200 mM pH 7.0 phosphate buffer was split into 666 wells (68.33 nmoles/well). To each well was added 1.2 equivalents of a tag (1 mM H_2_O, 82 µL) followed by 34.2 µL of a premixed solution of Cu(OAc)_2_ (2 eq, 10 mM DMF), TBTA (1 eq, 10 mM DMF), and sodium ascorbate (4 eq, 20 mM H_2_O). The reactions were allowed to proceed overnight and were precipitated and centrifuged the following day. The wells were redissolved in 68.33 µL of pH 9.5 borate buffer (500 mM). To each well was added 200 mM solutions of 222 bromo acryl carboxylic acids (34.2 µL, 100 eq), followed by 100 equiv. DMT-MM as a 200 µM aqueous stock. After overnight incubation the reactions were pooled and precipitated.

The pellet was redissolved in 46.52 mL of pH 9.5 buffer and any remaining amine was capped with 50 equivalents benzoic acid (11.6 mL, 200 mM DMA) and 40 equivalents of DMT-MM (4.65 mL, 400 mM H_2_O). Excess tags were capped using 1 equivalent of phenylacetylene (10 mM DMF, 2 mL) and 5 mL of the copper premix solution used in ligation. The library was then purified on reverse phase HPLC to yield 28 µmoles of material. The purified material was dissolved in 2.8 mL water, and 56 mL of TBAF solution (500 mM DMF) was added. After heating at 60° C for 1.25 hours, the DNA was precipitated, then desalted using centrifugal filtration.

# Cycle C

A 1 mM solution of Cycle A material (28 µmoles, 28 mL) in 200 mM pH 7.0 phosphate buffer was split into 669 wells (19 nmoles/well). To each well was added 1.3 equivalents of a tag (1 mM H_2_O, 24.7 µL) followed by 9.5 µL of a premixed solution of Cu(OAc)_2_ (2 eq, 10 mM DMF), TBTA (1 eq, 10 mM DMF), and sodium ascorbate (4 eq, 20 mM H_2_O). The reactions were allowed to proceed overnight and were precipitated and centrifuged the following day. The wells were redissolved in 19 µL of water, and 100 equivalents of Cs_2_CO_3_ (9.5 µL, 200 mM H_2_O) was added. This was followed by 80 equivalents of one of 667 boronic acids/esters (7.65 µL, 200 mM DMA) and 1.2 equivalents of Pd(PPh_3_)_4_ (2.28 µL, 10 mM DMA). The reactions were heated at 80° C for 3 hours, then were quenched with 31 equivalents of sodium diethyldithiocarbamate (3 µL/well, 200 mM H_2_O). The wells were pooled, precipitated and desalted by centrifugal filtration. Recovered 13 µmoles.

Excess tags were capped with .7 equivalents of phenylacetylene (10 mM DMF, .91 mL) and 1.63 mL of the copper premix solution used in ligation. The material was dissolved in 1.2 mL water, and 24 mL of TBAF solution (500 mM DMF) was added. After heating at 60° C for 1 hour, the DNA was precipitated, then purified on reverse phase HPLC to yield 4.7 µmoles of material.

Closing Tag Installation

10 nmoles of purified cycle C material was dissolved in 10 µL of 200 mM pH 7.0 phosphate, followed by addition of 2.5 equivalents of DNA closing tags (1 mM in H_2_O, 25 µL). To the DNA was added 5 µL of a premixed solution of Cu(OAc)_2_ (2 eq, 10 mM DMF, TBTA (1 eq, 10 mM DMF), and sodium ascorbate (4 eq, 20 mM H_2_O), and the reaction was allowed to proceed overnight. Upon completion, the wells were precipitated then treated with 50 equivalents of Na_2_S (400 mM H_2_O, 1.25 µL) at 50° C for 2 hours to precipitate any remaining copper, followed by desalting by centrifugal filtration.

**Affinity-mediated selection for sEH-binding small molecules**

Prior to selection the single-stranded library was converted to double-stranded form by hybridization of the closing tag to a complementary primer oligonucleotide and extension of this with the Klenow Fragment of DNA Polymerase I (exo-). The library (10 nmol), dNTPs (12 umol) and 400 ul of 10x NEB Buffer 2 were dissolved in water to a final volume of 3.6 ml. This solution was then heated at 65°C for 5 minutes, cooled to room temperature and 2000 units of the Klenow Fragment of DNA Polymerase I (exo-) (NEB) was then added and the mixture was incubated at 37°C for 48 hours with rotation. Subsequently the mixture was heated at 75°C for 20 minutes, lyophilized, re-dissolved in 400 ul of water, ethanol-precipitated twice with washing of the pellet in 70% ethanol, and re-dissolved in 67 ul of water for a presumed final concentration of 150 uM.

Affinity-based selection of sEH-binders was then initiated by dissolving the double-stranded closed library in a model cytosolic incubation buffer containing HEPES free acid (20 mM), potassium acetate (134 mM), sodium acetate (8 mM), sodium chloride (4 mM), magnesium acetate (0.8 mM), imidazole (5 mM), DTT (1mM), sheared salmon sperm DNA (1mg/ml, Invitrogen) and Tween-20 (0.02%) with pH adjusted to 7.2 with potassium hydroxide. To this solution was added His_6_-tagged Soluble Epoxide Hydrolase (Catalog number 10011669, Cayman Chemical) for a final concentration of 1 uM of sEH and 8.25 uM of library (assuming 100% yield of Klenow extension and work-up) in a final volume of 60 ul. The resultant mixture was incubated at room temperature for 1 hour and then the target and associated library members were captured on a 5ul bed of Ni-NTA-agarose capture matrix (His-Select^TM^ High-Flow Nickel Affinity Gel) constrained between two frits in a pipette tip (Phytip^TM^, Phynexus) over 20 minutes using a computer-controlled multi-channel pipetting system (ME-200^TM^, Phynexus). Washing was performed over 20 minutes with eight changes of the same buffer followed by elution at 85°C into the same buffer followed by incubation with a second 5ul bed of a Ni-NTA-agarose capture matrix to remove denatured sEH. Fresh sEH was then added to the flow-through and a second selection step was performed using the same protocol. Encoding oligonucleotides present in the second flow-through were amplified using Platinum PCR Supermix^TM^ (Invitrogen) with denaturation at 94°C, annealing at 55°C and extension at 72°C for 27 cycles using primer oligonucleotides (each at 0.5 uM) that incorporate complementary sequences to the tailpiece or headpiece complement along with the Illumina READ1 or READ2 sequences required to support clustering and subsequent single-read 100 base-pair sequencing on an Illumina HiSeq 2500.

**Sequencing results of naïve and sEH-selected library**

The number of sequence reads we generated from the naïve library was 2,037,412. The library contains 334 million encoded chemical entities and from a probabilistic perspective, if there were no biases in the library at all, 2,037,412 individual stochastic sampling events would be expected to observe the same triple building block combinations twice: 6,149 times, thrice: 12 times, and four times and above not at all (using the probability mass function and assuming a binomial distribution). Our sequencing data shows the same triple building block combinations twice: 66,492 times, thrice: 1,807 times, four times: 48, five times: once, and six times and above: not at all. This means that the most overrepresented building block triple combinations are observed about 14-fold over the average value for the naïve library and in turn suggests a very low level of bias. By comparison the building block combination that defines the active compound that we identified as having an IC50 value of 2 nM was observed 13 times out of a total of 147,885 qualifying sequence reads in the affinity-mediated selection output, which corresponds to an enrichment of 29,400-fold over the average for the naïve library. Also, as expected with a greater than 99% probability, this particular building block combination was not seen at all (zero times out of 2,037,412 sequence reads) in the naïve sequence dataset described above.

Expanded version of Figure 3. The vertical axis represents the number of observations of building block pairs, the size of points represents the number of observations of building block triples. Points are jittered by 0.5%.


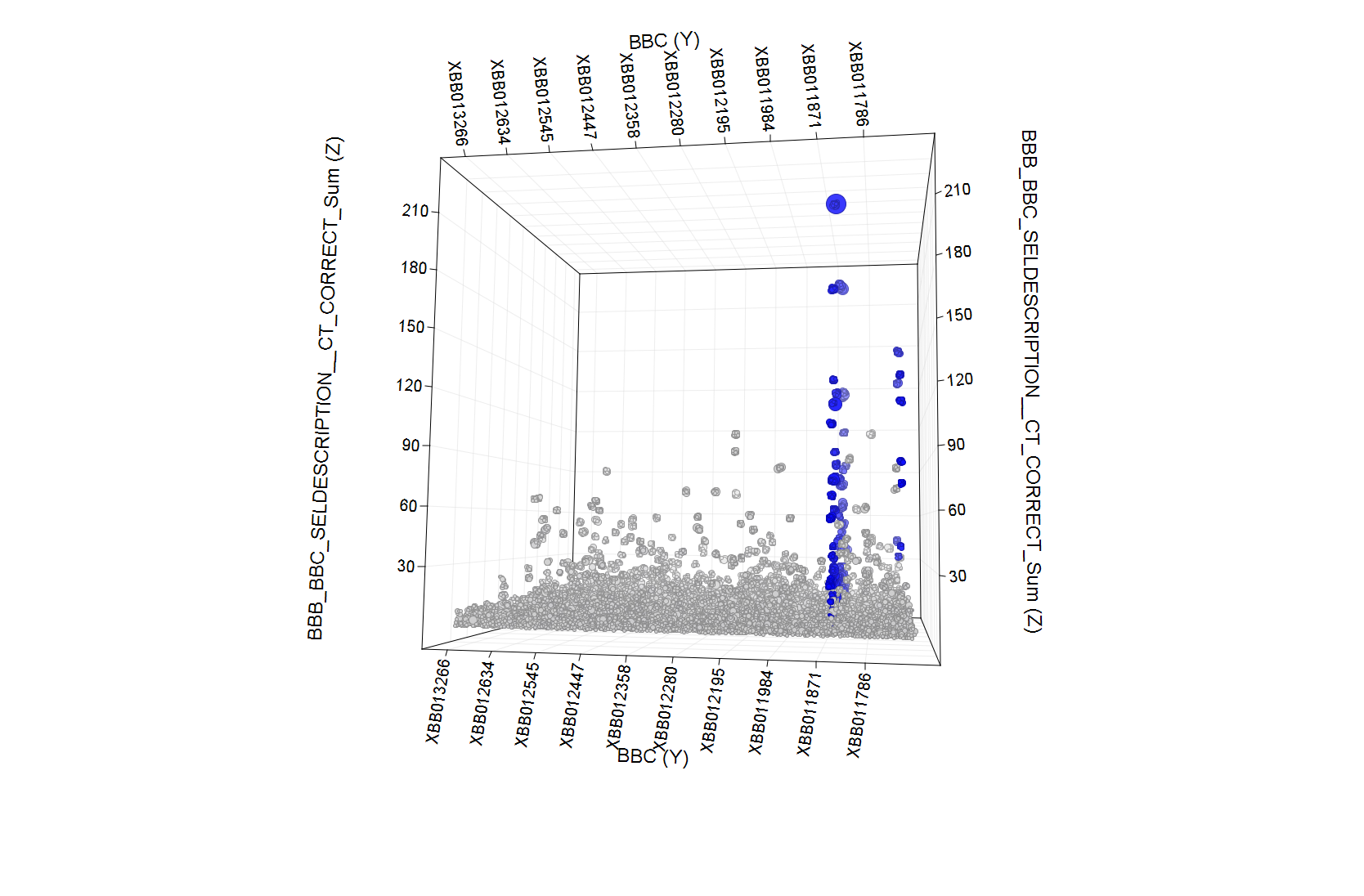


**Synthesis of Compound**

***3-(cyclohexylcarbamoyl)phenylboronic acid (S9)***

To a solution of **S8** (166 mg, 1 mmol) in DCM (10 mL) was added Bop-Cl (305 mg, 1.2 mmol) and DIEA (310 mg, 2.4 mmol) at r.t. After stirring for 0.5 h, cyclohexanamine (99 mg, 1 mmol) was added. The reaction mixture was stirred at RT for 4 hours. The resulting solution was diluted with DCM (20 mL), washed with 10% citric acid solution (2 x 15 mL), sat. NaHCO_3_ (2 x 15 mL), brine (20 mL), dried over Na_2_SO_4_ and filtered. The solvent was removed under reduced pressure to afford the crude product **S9** (207 mg, 83.8%) as a white solid. LC-MS (M+H)^+^ = 248.

***N-(2-(1H-pyrazol-1-yl)ethyl)-6-bromo-N-methylnicotinamide (S11)***

To a solution of **S10** (201 mg, 1 mmol) in DCM (10 mL) was added HATU (456 mg, 1.2 mmol) and DIEA (310 mg, 2.4 mmol) at RT. After stirring for 0.5 h, N-methyl-2-(1H-pyrazol-1-yl)ethanamine (125 mg, 1 mmol) was added. The reaction mixture was stirred at RT for 2 hours. The resulting solution was diluted with DCM (20 mL), washed with 10% citric acid solution (2 x 15 mL), sat. NaHCO_3_ (2 x 15 mL), brine (20 mL), dried over Na_2_SO_4_ and filtered. The solvent was removed under reduced pressure to afford the crude product **S11** (215 mg, 70%) as a yellow solid. LC-MS (M+H)^+^ = 309.

***N-(2-(1H-pyrazol-1-yl)ethyl)-6-(3-(cyclohexylcarbamoyl)phenyl)-N-methylnicotinamide (11)***

A mixture of **S9** (207 mg, 0.84 mmol), **S11** (215 mg, 0.7 mmol), Pd_2_(dba)_2_ (176 mg, 0.168 mmol), Xant-Phos (97 mg, 0.168 mmol) and Cs_2_CO_3_ (1.5 g, 14.1 mmol) in toluene (20 ml) was stirred at 90^o^C under N_2_ overnight. The resulting solution was diluted with EtOAc (40 mL) and filtered, the filtrate was concentrated under reduced pressure and purified by prep-HPLC to give **11** (150 mg, 50 %) as a white solid. LC-MS (M+H)^+^ = 432. ^1^H NMR (300 MHz, DMSO) δ 8.57 (s, 1H), 8.51 (s, 1H), 8.22 (d, 1H), 8.05 (s, 1H), 8.03 (d, 1H), 7.93 (d, 1H), 7.74 (s, 1H), 7.72 (s, 1H), 7.59 (t, 1H), 7.47 (s, 1H), 6.27 (s, 1H), 4.39 (t, 2H), 3.83 (m, 3H), 2.88 (s, 3H), 1.89-1.63 (m, 5H), 1.41 – 1.30 (m, 4H), 1.25 – 1.16 (m, 1H).

**Soluble Epoxide Hydrolase Fluorescent intensity assay**

Assays were conducted in costar 384 well black NBS plates (Corning). The assay buffer used was 25 mM Tris pH 7.0 and 0.1 mg/ml BSA. The enzyme was purchased from Cayman Chemical (10011669) at a stock concentration of 15.625 µM. The Epoxy Fluor 7 substrate also from Cayman Chemical (10008610) was dissolved in DMSO at a concentration of 25.68 mM. This was further diluted ten fold in DMSO to make 2.568 mM. Competition assays were performed with 5 µl Epoxy Fluor 7 diluted from DMSO stock to a final concentration of 5 nM, 5 µl of competitor (final concentration 10 µM −0.5 nM), and 10 µl enzyme (final concentration of 3 nM) in the assay buffer for a total volume of 20 µl. Compound was pre-incubated with enzyme for 15 min at RT prior to addition of substrate. The plate was read kinetically at excitation 330 nm and emission 465 nm wavelengths at 30 ^o^C for 20 minutes on the Tecan M1000. The rate was calculated for the linear portion of the curves (2.5-12.5 minutes) and data fit to a sigmoidal curve.
